# Blood Parasites in Endangered Wildlife-Trypanosomes Discovered during a Survey of Haemoprotozoa from the Tasmanian Devil

**DOI:** 10.3390/pathogens9110873

**Published:** 2020-10-23

**Authors:** Siobhon L. Egan, Manuel Ruiz-Aravena, Jill M. Austen, Xavier Barton, Sebastien Comte, David G. Hamilton, Rodrigo K. Hamede, Una M. Ryan, Peter J. Irwin, Menna E. Jones, Charlotte L. Oskam

**Affiliations:** 1 Centre for Biosecurity and One Health, Harry Butler Institute, Murdoch University, Murdoch, WA 6150, Australia; j.austen@murdoch.edu.au (J.M.A.); xavierbarton99@gmail.com (X.B.); p.irwin@murdoch.edu.au (P.J.I.); c.oskam@murdoch.edu.au (C.L.O.); 2 Department of Microbiology and Immunology, Montana State University, Bozeman, MT 59717, USA; m.ruiz.aravena@gmail.com; 3 School of Natural Sciences, College of Sciences and Engineering, University of Tasmania, Hobart, TAS 7001, Australia; sebastien.comte@dpi.nsw.gov.au (S.C.); d.g.hamilton@utas.edu.au (D.G.H.); rkhamede@utas.edu.au (R.K.H.); menna.jones@utas.edu.au (M.E.J.); 4 Vertebrate Pest Research Unit, NSW Department of Primary Industries, Orange, NSW 2800, Australia; 5 CANECEV, Centre de Recherches Ecologiques et Evolutives sur le Cancer (CREEC), 34090 Montpellier, France; 6 Health Futures Institute, Murdoch University, Murdoch, WA 6150, Australia

**Keywords:** Tasmanian devil, *Sarcophilus harrisii*, Haemoprotozoa, *Trypanosoma*, Marsupial, devil facial tumour disease (DFTD)

## Abstract

The impact of emerging infectious diseases is increasingly recognised as a major threat to wildlife. Wild populations of the endangered Tasmanian devil, *Sarcophilus harrisii*, are experiencing devastating losses from a novel transmissible cancer, devil facial tumour disease (DFTD); however, despite the rapid decline of this species, there is currently no information on the presence of haemoprotozoan parasites. In the present study, 95 Tasmanian devil blood samples were collected from four populations in Tasmania, Australia, which underwent molecular screening to detect four major groups of haemoprotozoa: (i) trypanosomes, (ii) piroplasms, (iii) *Hepatozoon*, and (iv) haemosporidia. Sequence results revealed *Trypanosoma* infections in 32/95 individuals. *Trypanosoma copemani* was identified in 10 Tasmanian devils from three sites and a second *Trypanosoma* sp. was identified in 22 individuals that were grouped within the poorly described *T. cyclops* clade. A single blood sample was positive for *Babesia* sp., which most closely matched *Babesia lohae*. No other blood protozoan parasite DNA was detected. This study provides the first insight into haemoprotozoa from the Tasmanian devil and the first identification of *Trypanosoma* and *Babesia* in this carnivorous marsupial.

## 1. Introduction

Haemoprotozoan parasites are unicellular eukaryotic organisms with complex lifecycles that involve an invertebrate vector and often alternate in their tropism between the tissues and blood of their vertebrate hosts. Four major haemoprotozoan assemblages infect mammals [1]; (i) Trypanosomatids (Kinetoplastea), which are flagellated protists that are characterised by the presence of a unique organelle, the kinetoplast; (ii) haemogregarines (Adeleorina); (iii) haemosporidia (Haemosporidia); and, (iv) piroplasms (Piroplasmida). Broadly haemoprotozoans are considered either host specific (e.g., *Theileria ornithorhynchi* infecting the platypus (*Ornithorhynchus anatinus*) host [2]) or generalists (e.g., *Trypanosoma cruzi*, which infects a wide range of mammals [3]). However, the true diversity and epidemiology of most of these species remain unknown.

The Tasmanian devil, *Sarcophilus harrisii*, is the largest extant marsupial carnivore. Once present across mainland Australia (~3000 years ago), the distribution of wild populations of Tasmanian devils became restricted to the island of Tasmania [4], off the southern coast of the Australian mainland. Biogeographical events have influenced declines in the effective size of devil populations and their distribution, with consequential genetic bottlenecks resulting in low genetic diversity [4,5,6]. Since 1996, a transmissible cancer (devil tumour facial disease, DFTD hereafter) gradually spread across most of the distributional range of the species and caused local population declines of upwards of 80% [7,8]. The transmission of DFTD occurs through direct transfer of live tumour cells between individuals when devils bite each other during social interactions [9,10,11]. Survival in DFTD infected individuals is usually 6–12 months after clinical signs appear; the disease is lethal in almost 100% of cases [12]. As a consequence of transmission being driven by social interactions, DFTD is still present, even in largely depleted populations of hosts [7]. Therefore, any additional pressure(s) on the host health, such as co-infections with haemoparasites, could further threaten imperilled populations, yet, little is known regarding the devil’s parasite community [13].

To date, only four protozoan parasite groups have been identified from wild devil populations; *Cryptosporidium* spp., *Giardia* spp., *Toxoplasma gondii*, and *Sarcocystis* sp. A previous study identified sporulated sporocysts consistent with *Sarcocystis* (family: Sarcocystidae) species from the intestinal mucosa using microscopic examination [14]. In north-west Tasmania, *T. gondii* prevalence was higher in carnivorous marsupials, such as the Tasmanian devil, spotted-tail quoll (*Dasyurus maculatus*), and eastern quoll (*D. viverrinus*), as compared to sympatric prey marsupials like the brushtail possum (*Trichosurus vulpecula*), Bennett’s wallaby (*Notamacropus rufogriseus*, syn. *Macropus rufogriseus*) and Tasmanian pademelon (*Thylogale billardierii*) [15]. Recently, *Cryptosporidium* and *Giardia* spp. were also identified from faecal samples of wild devils using molecular techniques [16].

Over the past two decades, the use of molecular techniques has significantly extended our understanding of haemoprotozoan parasites. As a result, it has revealed that Australian marsupials and monotremes can host a high diversity of haemoprotozoa species, most of which are unique to Australia [17,18,19,20]. The clinical effects of co-infections in wildlife are often difficult to evaluate. One well documented case is the investigation into a canine distemper virus epidemic in Serengeti lions (*Panthera leo*), which identified that joint infection by haemoprotozoa was a major contributing factor to fatal outcomes [21]. While there are limited studies on the clinical and pathological consequences of such interactions in native Australian wildlife, there is evidence that co-infection and co-morbidities can place species at an increased risk of disease when challenged with the infection of haemoprotozoa [22,23,24].

The trypanosomatids are obligatory parasites with a single flagellum and include several genera that are pathogenic to humans, animals, and plants [25,26]. Because of their medical importance, trypanosomatids have been studied more intensively with trypanosome and *Leishmania* parasites the causative agents of sleeping sickness (*Trypanosoma brucei gambiense* and *Trypanosoma brucei rhodesiense*), Chagas disease (*Trypanosoma cruzi*), and leishmaniases [26,27]. Broadly, *Trypanosoma* species can be divided into two groups based on their transmission route; the salivarian trypanosomes, which are transmitted via the saliva of the tsetse fly (*Glossina* spp.), and stercorarian trypanosomes, which are passed to their host via the faeces of the arthropod intermediate host [28]. The vectors of Australian trypanosomes are still largely unknown, with few studies investigating the presence of trypanosomes in invertebrates. Previous studies have implicated ticks (*Ixodes* spp.) [29,30] and tabanid flies [31] as potential vectors.

The present study aimed to screen blood samples from Tasmanian devils for the presence of haemoprotozoan parasites. To the authors’ knowledge, the present study provides the first survey of haemoprotozoa from Tasmanian devils.

## 2. Materials and Methods

### 2.1. Study Sites and Sampling

Animal use was approved by the University of Tasmania Animal Ethics Committee permit numbers A0015835 and A0016789) and the Department of Primary Industries, Parks, Water and Environment Animal Ethics Committee (permit numbers TFA19144 and TFA18028).

**Figure 1:**
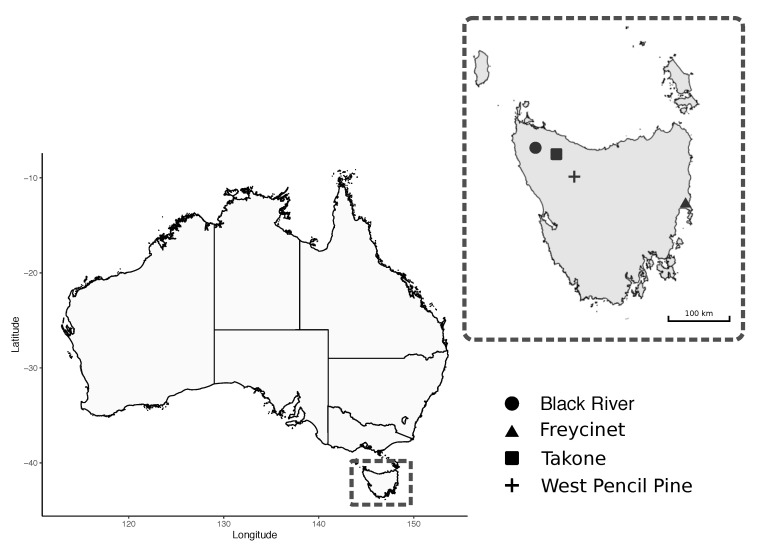
Map of study sites for field collection of Tasmanian Devil (*Sarcophilus harrisii*) samples.

A total of 95 blood samples were collected from wild Tasmanian devils that were captured during the austral autumn (May 2018) from four sites across Tasmania: Black River, Takone, West Pencil Pine and Freycinet (Figure 1). The sampling effort at each site involved deployment of 40 PVC pipe traps during seven to 10 consecutive nights. Individual devils were identified via subcutaneously implanted microchips (AllFlex© ISO FDX-B).

Blood was collected (between 0.3–1 mL) from either the jugular vein (Takone) or marginal ear vein (Black River, Freycinet and West Pencil Pine). The puncture site was disinfected with sterile alcohol swabs for at least 15 s prior to collection and blood was stored in ethylenediaminetetraacetic acid (EDTA) vacutainers. The samples were kept refrigerated at 4 ${}^{\circ }C$ and then shipped to Murdoch University, Western Australia where they were stored at −20 ${}^{\circ }C$ until analysis.

### 2.2. Molecular Screening

#### 2.2.1. DNA Extraction

The total genomic DNA was extracted from 200 μL of blood using a MasterPure DNA purification kit (Epicentre^®^ Biotechnologies, Madison, WI, USA) following the manufacturer’s recommendations. Where 200 μL of blood was not available, sterile DNA free phosphate-buffered saline (PBS) was used to make samples up to 200 μL. Genomic DNA (gDNA) was eluted in 30 μL of TE buffer and stored at −20 ${}^{{}^{\circ}}C$. Extraction controls (EXBs), consisting of 200 μL sterile DNA free PBS, were randomly included in each extraction batch (N = 7).

#### 2.2.2. PCR Assays

Molecular screening was carried out for haemoprotozoa on gDNA from blood samples (N = 95) and EXBs (N = 7). Reactions were carried out in 25 μL volumes containing 2 μL of gDNA or 1 μL of the primary product for nested and semi-nested PCR assays. Table 1 provides an overview of assays, including primer sequences.

For the identification of trypanosomes (*Trypanosoma* and *Leishmania* spp.) samples were screened using a nested PCR assay targeting an ~959 bp product of the second half of the 18S ribosomal RNA (18S rRNA) locus. This assay amplifies most species of *Trypanosoma* [32] and it was also validated in the present study to amplify *Leishmania* spp. using control gDNA of *Leishmania infantum* from a canine bone marrow sample and *Leishmania macropodum* culture isolate. External primers SLF/S762R and internal primers S825F/SLIR were used as an initial screen and then sequenced as per details below. Samples that were representative of the different genotypes identified then underwent an additional secondary assay with a second set of internal primers S823/S662R to yield a near full length 18S rDNA sequence. Reactions were carried out in 25 μL volumes containing 0.4 μM of each primer and 12.5 μL GoTaq^®^Green master mix (Promega, Madison, WI, USA). Thermal cycling conditions were as follows; initial cycle of 95 ${}^{{}^{\circ}}C$ for 5 min, 50 ${}^{{}^{\circ}}C$ for 2 min, 72 ${}^{{}^{\circ}}C$ for 4 min, followed by 35 cycles of 95 ${}^{{}^{\circ}}C$ for 30 s, 52 ${}^{{}^{\circ}}C$ (primary) or 55 ${}^{{}^{\circ}}C$ (secondary) for 30 s, 72 ${}^{{}^{\circ}}C$ for 2 min. 20 s (primary) or 1 min. (secondary), and a final extension of 72 ${}^{{}^{\circ}}C$ for 5 min. To obtain further phylogenetic information from the genotypes that were identified by 18S rRNA locus screening, samples underwent amplification of the glycosomal Glyceraldehyde Phosphate Dehydrogenase (*gGAPDH*) gene using a semi-nested PCR assay with external primers GAPDHF/GAPDHR and internal primers GAPDHF/Ga4 [32]. Reactions contained 1X buffer (KAPA Biosystems, Cape Town, South Africa), 2.0 mM (MgCl${}_{2}$), 0.4 μM of each primer, 0.25 mM of each dNTP, and 0.5 U of Taq (KAPA Biosystems, Cape Town, South Africa). Thermal cycling conditions were as follows; initial cycle of 95 ${}^{{}^{\circ}}C$ for 5 min, 50 ${}^{{}^{\circ}}C$ for 2 min, 72 ${}^{{}^{\circ}}C$ for 4 min, followed by 35 cycles of 95 ${}^{{}^{\circ}}C$ for 30 s, 52 ${}^{{}^{\circ}}C$ (primary) or 55 ${}^{{}^{\circ}}C$ (secondary) for 30 s, 72 ${}^{{}^{\circ}}C$ for 2 min. 20 s, and a final extension of 72 ${}^{{}^{\circ}}C$ for 5 min.

Screening for piroplasms (*Babesia* and *Theileria* spp.) utilised a previously described nested PCR assay. Primers were used targeting an ~800 bp product of the 18S rRNA locus with external primers BT1F/BT1R and internal primers BT2F/BT2R [35]. The reactions contained 1× buffer (KAPA Biosystems, Cape Town, South Africa), 2.0 mM (MgCl${}_{2}$), 0.4 μM of each primer, 0.25 mM of each dNTP, and 0.5 U of Taq (KAPA Biosystems, Cape Town, South Africa). Thermal cycling conditions were as follows; initial cycle of 95 ${}^{{}^{\circ}}C$ for 2 min, 58 ${}^{{}^{\circ}}C$ for 1 min, 72 ${}^{{}^{\circ}}C$ for 2 min, followed by 35 cycles of 95 ${}^{{}^{\circ}}C$ for 30 s, 62 ${}^{{}^{\circ}}C$ for 20 s, 72 ${}^{{}^{\circ}}C$ for 45 s, and a final extension of 72 ${}^{{}^{\circ}}C$ for 7 min.

A genus specific assay for *Hepatozoon* targeting the 18S rRNA locus was used with primers HepF300/Hep900 in order to amplify an ~600 bp product [36]. Reactions contained 1× buffer (KAPA Biosystems, Cape Town, South Africa), 1.5 mM (MgCl${}_{2}$), 0.4 μM of each primer, 0.25 mM of each dNTP, and 0.5 U of Taq (KAPA Biosystems, Cape Town, South Africa). Thermal cycling conditions were as follows; initial denaturation of 95 ${}^{{}^{\circ}}C$ for 3 min, followed by 40 cycles of 95 ${}^{{}^{\circ}}C$ for 30 s, 60 ${}^{{}^{\circ}}C$ for 30 s, 72 ${}^{{}^{\circ}}C$ for 1 min, and a final extension of 72 ${}^{{}^{\circ}}C$ for 10 min.

Screening for haemosporidia (*Plasmodium* spp.) utilised a previously described nested PCR assay [37]. Primers targeting an ~420 bp product of the *cytochrome b* (*cytb*) gene with external primers HAEMNF/HAEMNR2 and internal primers HAEMF/HAEMR2. Reactions contained 1× buffer (KAPA Biosystems, Cape Town, South Africa), 1.5 mM (MgCl${}_{2}$), 0.4 μM of each primer, 0.25 mM of each dNTP, and 1.0 U of Taq (KAPA Biosystems, Cape Town, South Africa). The thermal cycling conditions were as follows; initial denaturation of 95 ${}^{{}^{\circ}}C$ for 8 min, followed by 35 cycles of 95 ${}^{{}^{\circ}}C$ for 30 s, 50 ${}^{{}^{\circ}}C$ (primary) or 52 ${}^{{}^{\circ}}C$ (secondary) for 30 s, 72 ${}^{{}^{\circ}}C$ for 45 s, and a final extension of 72 ${}^{{}^{\circ}}C$ for 10 min.

Suitable positive controls, extraction reagent blanks, and no-template PCR controls were included throughout the laboratory processes. Extractions, pre-PCR and post-PCR procedures were performed in laboratories physically separated from each other.

#### 2.2.3. Gel Electrophoresis and Sanger Sequencing

Amplicons were electrophoresed on a 1% agarose gel stained with SYBR safe (Invitrogen, Waltman, MA, USA). Products of the correct size (see Table 1) were excised from the gel and purified while using the previously described methods [39]. Sanger sequencing was carried out in forward and reverse directions on all positive amplicons. Sequencing was performed at the Australian Genome Research Facility (Perth, Western Australia) on an Applied Biosystems 3730 using Big Dye Terminator chemistry version 3.1.

#### 2.2.4. Phylogenetic Analysis

The sequences were imported and trimmed in Geneious 10.2.6 (https://www.geneious.com) and then subjected to BLAST analysis using BLASTN 2.10.0+ [40] against nucleotide collection (nt) database [41] to identify the most similar species and genotypes. Reference sequences were retrieved from GenBank [42] (details available in Supplementary Table S1) and aligned with sequences obtained in the present study using MUSCLE [43]. Alignments of the 18S rDNA and the gGAPDH sequences were then used for phylogenetic purposes. Phylogenies were inferred while using the maximum likelihood (ML) method. The optimal evolutionary model was selected while using ModelFinder [44] based on bayesian information criterion. Phylogenetic analysis was performed in IQ-TREE v1.6.11 [45] and bootstrap support was calculated using the ultrafast (UFBoot2) method with 10,000 replicates [46].

Maximum likelihood phylogeny based on the 18S rRNA locus was performed using a 1505 bp alignment based on the transition (AC = CG, AT = GT with equal base frequencies) (TIM3e) substitution model [47], with invariable sites (I = 0.408) and a discrete gamma distribution (four categories) (G4 = 0.438) [48]. To include a wider range of neighbouring reference sequences, a second phylogenetic analysis was performed on the V7–8 hypervariable region of the 18S rRNA locus; this region is useful to differentiate between closely related trypanosome sequences [49]. A phylogeny was produced using a 559 bp alignment that was based on the Kimura Two-Parameter (K2P) substitution model [50] with discrete gamma distribution (four categories) (G4 = 0.227) [48]. A 767 bp alignment of the *gGAPDH* gene was used for phylogenetic reconstruction based on the Tamura-Nei (TN) substitution model [51], with empirical base frequencies (F), invariable sites (I = 0.387) and discrete gamma distribution (four categories) (G4 = 0.893) [48]. Genetic sequence similarity was calculated while using the Kimura Two-Parameter method [51].

Sequences that were generated in the present study have been submitted to GenBank nucleotide database under accession numbers MT883295–MT883326 (*Trypanosoma* 18S rDNA), MT514664–MT514666 (*Trypanosoma* GAPDH), and MW084364 (*Babesia* 18S rDNA).

### 2.3. Statisical Analysis

The overall prevalence of *Trypanosoma* species was compared between males and females while using two-tailed Fisher Exact test.

### 2.4. Microscopy of Blood Smears

Thin blood smears were prepared from animals that were sampled at Takone site only (three per individual), within 4 h of collection, and then air dried and fixed in methanol. Blood smears were then stained with modified Wright-Giemsa (Hematek^®^Stain Pak) using a Hema-Tek Slide Stainer (Ames Company Division, Miles Laboratories Pty Ltd., Victoria, Australia) and a coverslip was mounted using DPX neutral mounting medium (LabChem, Victoria, Australia). The smears were inspected by light microscopy (Olympus BX51) for the presence of haemoprotozoa at ×400 magnification and under oil immersion (×1000).

## 3. Results

### 3.1. Molecular Screening

Molecular screening identified 33.7% (32/95) of the blood samples were positive for *Trypanosoma* DNA. Sequence identity from BLAST results identified; *T. copemani* and *T. cyclops*-like in 10.5% (*n* = 10) and 23.2% (*n* = 22) of the individuals respectively (Table 2). *Trypanosoma copemani* was almost exclusively identified in males (90.0%, *p* = 0.0159), while *T. cyclops*-like genotypes were more commonly found in females (68.2%, *p* = 0.0432). All of the samples tested negative for presence of *Leishmania*, *Hepatozoon*, *Plasmodium*, and *Theileria* species.

**Figure 2:**
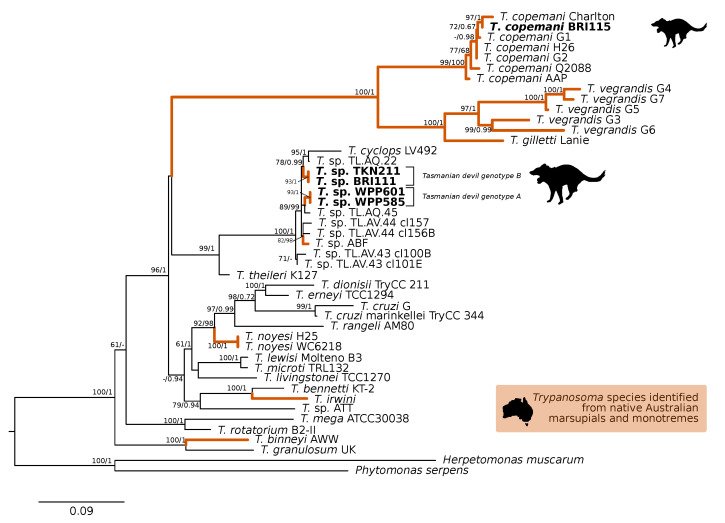
Maximum likelihood (ML) phylogenetic reconstruction of the *Trypanosoma* genus based on a 1505 bp alignment of the 18S rRNA locus using TIM3e + I + G4 substitution model. The node values correspond to bootstrap support/aBayes support, values less than 60 are hidden. Number of substitutions per nucleotide position is represented by the scale bar. Lineages that have been described from native Australian marsupials are denoted by orange lines. Sequences that were generated in the present study in bold. GenBank accession numbers for sequences are available in Supplementary Table S1.

**Figure 3:**
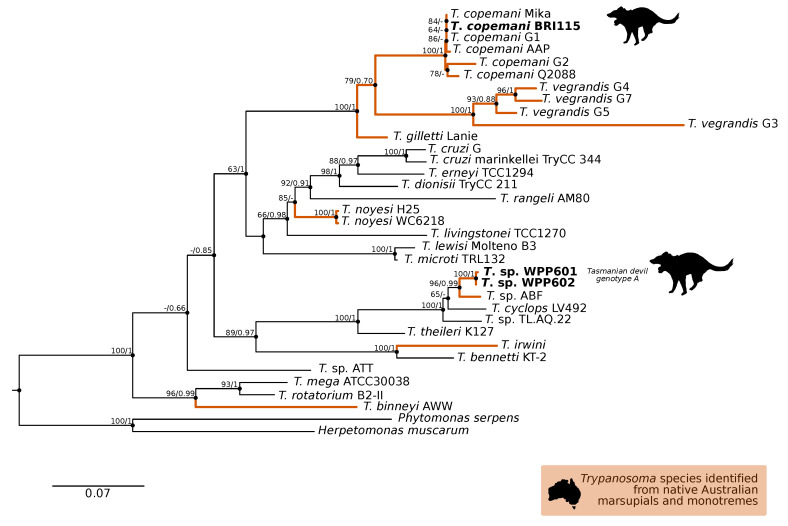
Maximum likelihood (ML) phylogenetic reconstruction of the *Trypanosoma* genus based on a 767 bp alignment of glycosomal Glyceraldehyde Phosphate Dehydrogenase (gGAPDH) while using TN93 + F + I + G4 substitution model. The node values correspond to bootstrap support/aBayes support, values less than 60 are hidden. Number of substitutions per nucleotide position is represented by the scale bar. Lineages that have been described from native Australian marsupials are denoted by orange lines. Sequences generated in the present study are in bold. GenBank accession numbers for sequences are available in Supplementary Table S1.

**Figure 4:**
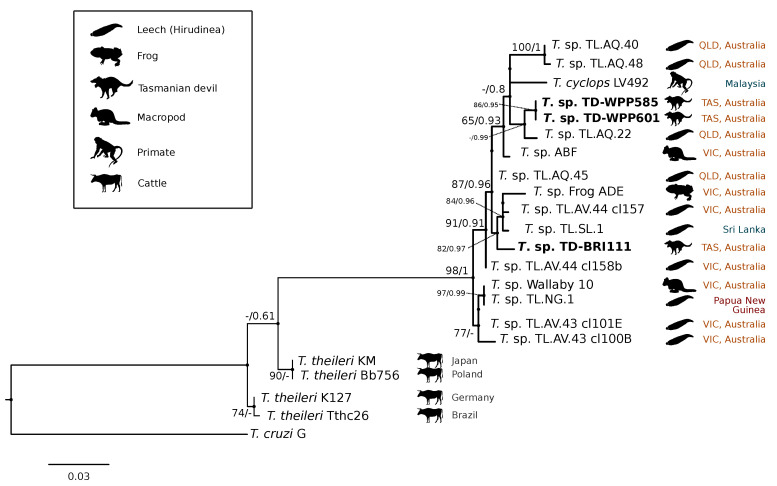
Maximum likelihood (ML) phylogenetic reconstruction of sequences from the *Trypanosoma cyclops* clade based on a 559 bp alignment of the 18S rRNA locus across the V7-8 hypervariable region while using K2P + G4 substitution model. Node values correspond to bootstrap support/aBayes support, values less than 60 are hidden. The number of substitutions per nucleotide position is represented by the scale bar. Australian states are abbreviated to QLD (Queensland), TAS (Tasmania), and VIC (Victoria). GenBank accession numbers for sequences are available in Supplementary Table S1.

A comparison at the 18S V7-8 hypervariable region of the ten *T. copemani* sequences showed they were all >99% similar to each other and, as such, a representative sample (BRI115) was used for subsequent phylogeny. Analysis of long 18S alignment concluded that the BRI115 sample shared 99.1% similarity to *T. copemani* Charlton (GU966588) and 98.5% with *T. copemani* H26 (AJ009169) (Figure 2). At the gGAPDH locus BRI115 sample was identical to *T. copemani* Mika (GU966585), and 99.7% similar to *T. copemani* AAP (AJ620277) (Figure 3). There is no 18S rDNA sequence data for *T. copemani* Mika available. A comparison of *T. copemani* Charlton (GU966584) at the gGAPDH locus showed it was 99.9% similar to the BRI115 sample with one single nucleotide polymorphism (SNP) (data not shown). At the 18S V7-8 hypervariable region two genotypes of the *T. cyclops* clade were identified, referred to as Tasmanian devil genotype A (*n* = 13) and genotype B (*n* = 9). Representatives of each genotype were used for phylogenetic reconstruction that was based on a longer alignment of 18S rDNA sequences (Figure 2). Analysis of near full length 18S rDNA alignment showed sequences were identical within each genotype (genotype A samples WPP585 and WPP601, and genotype B samples TKN211 and BRI111). The most similar 18S sequences to genotype A were *Trypanosoma* sp. TL.AQ.22 (AJ620574; 98.8% similar) and *T. cyclops* (AJ131958; 96.9% similar). Genotype B 18S sequences were most similar to *Trypanosoma* sp. ABF (AJ620564; 99.2% similar) and *Trypanosoma* sp. TL.AV.43 cl.101E (AJ620571; 99.1% similar). The gGAPDH sequences that were obtained from genotype A samples WPP601 and WPP602, were 99.9% similar to each other (Figure 3), with just one SNP. The most similar sequence was *Trypanosoma* sp. ABF (AJ620278), which was 97.2% and 97.4% similar to WPP601 and WPP602, respectively. The next most similar sequence was *T. cyclops* (FJ649493), which was 95.7% and 95.8% similar to WPP601 and WPP602, respectively. Unfortunately, GAPDH sequences from genotype B were not successfully amplified. Phylogenetic reconstruction utilising the V7-8 hypervariable region of the 18S rRNA locus showed support for a monophyletic *T. cyclops* clade (Figure 4). Sequences from the present study grouped with genotypes detected from Malaysia, Sri Lanka and Australia (Queensland and Victoria) with the clear distinction of two different genotypes of *T. cyclops* from Tasmanian devils (Supplementary Table S2).

A single sample (BRI115) obtained from Black River site was positive for a *Babesia* sp. and this individual was also positive for *T. copemani* infection. Analysis of an 800 bp fragment of the 18S rRNA locus revealed it was similar to *Babesia* sp. identified from *Ixodes tasmani* in Queensland (MG251436; 96.0% similarity) and *Babesia lohae* from *Ixodes holocyclus* in Queensland (MG593272; 95.5% similarity).

### 3.2. Microscopy

Microscopy of blood smears from Takone site did not yield any positive detection of haemoparasites.

## 4. Discussion

The present study represents the first survey of haemoprotozoa from wild populations of the endangered Tasmanian devil. Our findings of *Trypanosoma* infections across all four sites suggests that this infection is widespread and potentially endemic within Tasmanian devil populations. This wide distribution of *Trypanosoma* across populations contrasts with the absence of *Leishmania*, *Theileria*, *Hepatozoon*, or *Plasmodium* species, and the low detection of *Babesia*, which was only detected in a single individual.

The extension of the host range of *T. copemani* is notable and it supports recent research identifying this parasite in a wide range of marsupial hosts from across Australia. To date, *T. copemani* has been identified in all Australian states and territories, except the Northern Territory and South Australia. The identification of this parasite in Tasmanian devils is potentially significant from a health perspective as previous reports have associated it with pathological changes in the woylie (*Bettongia penicillata*) [22,23] and in koalas (*Phascolarctos cinereus*) with co-morbidities [24]. With this report, there are now at least ten Australian vertebrate host records for *T. copemani*; therefore, this *Trypanosoma* species appears to be a marsupial generalist and capable of infecting a diverse range of mammals. Further studies for mapping the complete distribution and host range of *T. copemani* will help to provide insights into the co-evolution of this native trypanosome in its marsupial hosts.

The identification of genotypes from the *Trypanosoma cyclops* clade was unexpected. *Trypanosoma cyclops* was described from wild caught southern pig-tail macaques (*Macaca nemestrina*) in jungle areas of West Malaysia [52] and Hamilton et al. [53] identified novel genotypes of this clade from Australia, Papua New Guinea, and Sri Lanka. However, since its identification, there have been very few published reports of *T. cyclops*. Phylogeny within the *T. cyclops* clade was not well resolved, as demonstrated by the polytomy in Figure 4. The identification of two distinct genotypes in devils that do not form a monophyletic group and show different levels of relatedness to sequences overseas highlights the uncovered diversity within this clade. The closest named species to *T. cyclops* is *Trypanosoma* (*Megatrypanum*) *theileri*, which has a cosmopolitan distribution and predominantly infects cattle. The most important vector of *T. theileri* is thought to be tabanid flies (Tabanidae) [54,55]. While this trypanosome is generally considered non-pathogenic, chronic infection has been associated with the development of secondary diseases in cattle [56].

The *gGAPDH* gene has been shown to be a more suitable marker than the 18S rRNA locus for determining the phylogeny within the *Trypanosoma* genus [34]. A comparative analysis of *T. copemani* shows the intra-specific sequence similarity within the clade is 97.0–100% and inter-specific similarity to the nearest named species, *T. gilletti*, is 91.3–92.3%. Intra-specific sequence similarity within the *T. cyclops* clade was 94.8–99.9%, while the inter-specific sequence similarity to nearest named species, *T. theileri*, is 90.4%. Therefore, there is sufficient support that the sequences generated for devils can be attributed to *T. cyclops*. The differentiation of genotypes within this clade are best identified by analysis of the V7-8 hypervariable region of the 18S rRNA locus (Figure 4, Supplementary Table S2). This study further supports that *T. cyclops* is more closely related to sequences obtained from Australia, Papua New Guinea, and Sri Lanka than it is to *T. theileri*. [53]. There is no geographical or host distinction between the different genotypes, further supporting the taxonomy as a single species, as shown in the phylogeny within the *T. cyclops* clade (Figure 4). Research on functional traits, including growth dynamics in culture coupled with morphological analysis, is needed to better understand the taxonomy of this clade. The geographical and host extensions reported here will be useful for the future work that aimed at understanding the evolutionary history of the *T. cyclops* and *T. theileri* clades and the *Trypanosoma* genus more broadly [57].

The description of a *T. cyclops*-like species in this work expands the number of *Trypanosoma* species in Tasmanian mammals to four. The platypus-specific *T. binneyi* has been observed from blood samples and subsequently described phylogenetically [29,58,59] with suggestions that leeches are the likely vector [59]. Trypanosomes have also been identified in populations of the eastern barred bandicoot (*Perameles gunnii*) and southern brown bandicoot (*Isoodon obesulus*) [60]; however, due to well-described limitations of morphological identification [61], species-level identification has not been achieved. Additionally, *T. copemani* was identified for the first time in Tasmania in 2020 from wild populations of the eastern quoll (*Dasyurus viverrinus*) [62]. The current study now brings the number of known trypanosome hosts in Tasmania to five, with the addition of Tasmanian devils, and the confirmation of a new species from the *T. cyclops* clade. It is interesting to note that, while previous studies have only described one species of *Trypanosoma* in the sampled hosts from Tasmania, in the current study we have described at least two species circulating in devils. The higher diversity of *Trypanosoma* species found in devils might be a consequence of our larger sample size or spatial coverage in comparison to previous studies.

Despite the molecular identification of *Trypanosoma* infection in devils from Takone, no trypomastigotes were observed in corresponding blood smears. This finding is consistent with other studies that have found molecular tools are more sensitive than microscopy for trypanosome detection [63,64]. The absence of patent parasitaemia (i.e. no flagellates observed in fresh blood smears) could be the result of low-level infections; however, it may also indicate that devils are not a competent host of the *Trypanosoma* species [65].

The phylogenetic position of the *T. cyclops* clade within the stercorarian group means that its potential vector(s) could be a range of arthropod(s) with transmission occurring via contact with vector faeces. While it has been hypothesised that carnivory is important in the maintenance of trypanosomes, experimental studies do not support this [66] and, instead, suggested that increased infection is attributed to insectivores who consume infected arthropod vectors [67]. However, it is interesting that, although members of this clade have been identified from leeches and frogs [53], their phylogenetic position is distinct from other aquatic and leech-associated *Trypanosoma* species [34,59,68,69]. The range extensions of both *T. copemani* and *T. cyclops* clades that were recorded in the present study provide additional data to aid vector identification. The large geographical range of both these *Trypanosoma* species suggests that any vector(s) should be equally ubiquitous and capable of biting or coming into close contact with a wide range of vertebrate hosts.

In the present study, we validated that a previously published *Trypanosoma* PCR assay [32] is also capable of detecting/amplifying *Leishmania* DNA. Two controls of genetically diverse species of *Leishmania*, *L. infantum* and *L. macropodum*, [70] were used. In the context of this study, we determined that generic trypanosome primers are able to amplify *L. macropodum*, which was identified as a cause of cutaneous leishmaniasis in kangaroos [71]. The preferred diagnostic sample for the detection of *Leishmania* is generally bone marrow aspirate or tissue samples; however, previous studies have recorded detection via blood samples [72,73]. Obtaining bone marrow and internal tissue samples from free ranging wildlife is not practical or ethical, especially where populations are experiencing significant declines. While the present study reported the absence of *Leishmania* via PCR of blood samples, it is not possible to discount occult infection and future studies utilising additional sample types would be recommended. Although co-infections of *Leishmania* and *Trypanosoma* cannot be completely ruled out in the individuals tested here, the absence of *Leishmania* DNA is consistent with previous studies that have utilised the same assay on other Australian mammals [17,18,74]. Therefore, reports of Australia’s only endemic *Leishmania* species remain confined to kangaroos and biting midges (Ceratopogonidae) in the Northern Territory [71,75,76].

An unexpected outcome was the low detection of *Babesia* species and lack of detection of *Theileria* and *Hepatozoon* species. Recent molecular studies in Australian wildlife have demonstrated a range of unique endemic piroplasms, with reports of up to 80–90% prevalence in some marsupial host populations [2,19,77]. Piroplasms and *Hepatozoon* spp. are transmitted by ticks and, therefore, dependent on the density and interactions between host and vector(s) to continue their lifecycle. Recent investigations have revealed the presence of numerous novel *Babesia*, *Theileria*, and *Hepatozoon* species in native Australian ticks [78,79,80]. With respect to the single identification of *Babesia* at the Black River site, the genetic relationship shows that it is most closely related to *Babesia lohae* sequences identified in *Ixodes holocyclus* and *Ixodes tasmani* ticks from the east coast of Queensland [78,79]. The low prevalence of *Babesia* might be attributed to the seasonality of tick infestations, as has been described in the relationship between *I. tasmani* ticks and brushtail possums hosts [81]. Future longitudinal sampling, including multiple seasons, are valuable to better understanding the phylogeny and epidemiology of this *Babesia* species circulating in devil populations.

In Tasmania, piroplasms and *Hepatozoon* species have been identified from three marsupial species and, more recently, from ticks (*Ixodes* spp.). A *Hepatozoon* has been identified in eastern barred bandicoots (*Perameles gunnii*) [82] by morphological examination of blood smears and the eastern quoll (*Dasyurus viverrinus*) through molecular tools [62]. *Theileria* species have been identified from eastern bettongs (*Bettongia gaimardi*) [83] and eastern quolls [62]. A molecular survey of ticks (*I. tasmani*) from Tasmanian devils demonstrated that 34.1% (15/44) of sample pools were positive for *Hepatozoon* sp. [84]. More recently, novel species of *Theileria* and *Hepatozoon* were identified from *I. tasmani* collected in Tasmania [78,79]. Given the high prevalence of *I. tasmani* parasitising sampled Tasmanian devils (Ruiz-Aravena pers. comms.), the low prevalence of piroplasms and *Hepatozoon* species is, therefore, unexpected. This raises important questions about the sylvatic lifecycle and reservoir hosts of these piroplasm and *Hepatozoon* species, suggesting potential and contrasting explanations: (i) Tasmanian devils are not competent reservoir hosts or (ii) they are natural hosts which are able to mount a sufficient immune response against the infection. In both cases, parasitaemia might be low or absent. Additional explanations could include (iii) infections by haemoprotozoa are acute and fatal to Tasmanian devils; however, the absence of *Theileria* and *Hepatozoon* in DFTD-free individuals makes this hypothesis less likely; or, (iv) the detection of haemoprotozoa in ticks is an opportunistic/accidental finding and they are not competent vectors of these organisms. Additional sampling from DFTD-free populations and capturing seasonal variation will be important for assessing the prevalence and susceptibility of Tasmanian devils to these haemoprotozoans.

The inclusion of morphological data obtained from blood smears and the application of additional molecular tools, such as next-generation sequencing to identify co-infections [85] and genome level information [86], are vital for further work. The collection of additional tissue samples may also prove to be useful in developing diagnostic tools, as well as better understanding the lifecycle of haemoprotozoa in the host. For example, bone marrow, skeletal muscle, tongue, brain, and liver samples have been tested positive for *Trypanosoma* DNA, while blood samples (including blood smears) were negative [87]. While there is a body of work demonstrating that Australian mammals are hosts to a range of endemic species of *Trypanosoma*, *Leishmania*, *Babesia*, *Theileria*, and *Hepatozoon* [17,18,19,31], the clinical impact of these haemoprotozoa is largely unknown. This is particularly important in the context of endangered species, such as the Tasmanian devil.

The results that are presented here highlight the need for additional studies on the haemoprotozoa and broader parasite community of Tasmanian devils. It is vital that the complete biology of this species be understood in order to provide meaningful information for management and conservation strategies. Conservation strategies, including translocation, captive breeding, and establishment of insurance populations, are management strategies that have been implemented in the case of Tasmanian devils. Parasite communities are an important consideration in species recovery, highlighting the need to further study host-parasite relationships [88]. For example, while the translocation of animals could facilitate introduction of parasites into new host populations, the same translocation could dilute adaptations of the receiving populations to the local parasites. This last case is particularly relevant when the local individuals in supplemented populations are outnumbered by the translocated individuals. Immediate future work is now needed in order to document the prevalence and diversity of haemoprotozoa in devils with a focus towards understanding the clinical impacts of infection and importantly impact of co-infection with DFTD.

## 5. Conclusions

Here, we have provided the first insights into haemoprotozoa infecting the endangered Tasmanian devil. Further research is urgently needed in order to document the full haemoprotozoan diversity in the Tasmanian devil, with a focus towards understanding the clinical significance of these infections and co-infections with DFTD. The identification of *T. copemani* and novel members of the *T. cyclops* clade provide further insights into the co-evolution of trypanosomes in marsupials, showing that Australian native fauna harbour genetically unique parasitic species. Finally, the lack of detection of major haemoprotozoa genera; *Hepatozoon*, *Leishmania*, *Plasmodium*, and *Theileria* in our study opens questions regarding the overall status of these parasites in the island ecosystem of Tasmania and whether their absence might reflect specific host-parasite interactions.

## Figures and Tables

**Table 1:** List of primers used for screening of haemoprotozoa from Tasmanian Devils (*Sarcophilus harrisii*).

Target	Primer	Sequence	Size	Ref.
Trypanosomes				
18S primary SSU	SLF	GCTTGTTTCAAGGACTTAGC	~1.5 kb	[32]
	S762R	GACTTTTGCTTCCTCTAATG		[33]
18S secondary SSU2	S823F	CGAACAACTGCCCTATCAGC	~904 bp	[33]
	S662R	GACTACAATGGTCTCTAATC		[33]
18S secondary SSU1	S825F	ACCGTTTCGGCTTTTGTTGG	~959 bp	[33]
	SLIR	ACATTGTAGTGCGCGTGTC		[32]
GAPDH primary	GAPDGF	CTYMTCGGNAMKGAGATYGAYG	~900 bp	[32]
	GAPDHR	GRTKSGARTADCCCCACTCG		[32]
GAPDH secondary	GAPDGF	CTYMTCGGNAMKGAGATYGAYG	~880 bp	[32]
	Ga4	GTTYTGCAGSGTCGCCTTGG		[34]
Piroplasms				
18S primary	BT1F	GGCTCATTACAACAGTTATAG	~903 bp	[35]
	BT1R	CCCAAAGACTTTGATTTCTCTC		[35]
18S secondary	BT2F	CCGTGCTAATTGTAGGGCTAATAC	~800 bp	[35]
	BT2R	GGACTACGACGGTATCTGATCG		[35]
*Hepatozoon* spp.				
18S	HepF300	GTTTCTGACCTATCAGCTTTCGACG	~600 bp	[36]
	Hep900	CAAATCTAAGAATTTCACCTCTGAC		[36]
Haemosporidia				
*Cytb* primary	HaemNF	CATATATTAAGAGAATTATGGAG	~580 bp	[37]
	HaemNR2	AGAGGTGTAGCATATCTATCTAC		[37]
*Cytb* secondary	HaemF	ATGGTGCTTCGATATATGCATG	~520 bp	[38]
	HaemR2	GCATTATCTGGATGTGATAATGGT		[38]

**Table 2:** Overview of Tasmanian devil sampling across survey sites showing number of individuals samples (n) and number of individuals positive for *Trypanosoma* species.

Site	*n*	*Trypanosoma* spp.	*T. copemani*	*T. cyclops*-Like
Black River	31	11	2	9
Takone	23	7	5	2
West Pencil Pine	14	11	0	11
Freycinet	27	3	3	0
Overall	95	32	10	22

## Supplementary Materials

The following are available online at https://www.mdpi.com/2076-0817/9/11/873/s1. Supplementary Table S1. *Trypanosoma* sequences used in phylogenetic analysis in the present study. Supplementary Table S2. Pairwise genetic similarity matrix of the *Trypanosoma cyclops* clade at the 18S rRNA locus. Analysis conducted over at 559 bp alignment of the V7-8 hypervariable using the Kimura Two-Parameter (K2P) method.

## Abbreviations

The following abbreviations are used in this manuscript:

18S rDNA	18S ribosomal DNA
DFTD	Devil tumour facial disease
gDNA	genomic DNA
gGAPDH	glycosomal glyceraldehyde phosphate dehydrogenase gene
SNP	single nucleotide polymorphism
