# Supplementary material for: Blood Parasites in Endangered Wildlife-Trypanosomes Discovered during a Survey of Haemoprotozoa from the Tasmanian Devil
Source: Pathogens. 2020 Oct 23;9(11):873. doi: 10.3390/pathogens9110873 (PMC7690708; doi:10.3390/pathogens9110873)
Supplement: Supplementary file 1 [file pathogens-09-00873-s001.zip › Supplementary_Tables/Supplementary_table_A2.docx]

**Supplementary Table A2.** Pairwise genetic similarity matrix of the *Trypanosoma cyclops* clade at the 18S rRNA locus. Analysis conducted over at 559 bp alignment of the V7-8 hypervariable using the Kimura Two-Parameter (K2P) method.

|  | 1 | 2 | 3 | 4 | 5 | 6 | 7 | 8 | 9 | 10 | 11 | 12 | 13 | 14 | 15 | 16 | 17 | 18 | 19 | 20 | 21 |
| --- | --- | --- | --- | --- | --- | --- | --- | --- | --- | --- | --- | --- | --- | --- | --- | --- | --- | --- | --- | --- | --- |
| *T. theileri* KM AB007814 |  |  |  |  |  |  |  |  |  |  |  |  |  |  |  |  |  |  |  |  |  |
| *T. theileri* Bb756 KF765801 | 100 |  |  |  |  |  |  |  |  |  |  |  |  |  |  |  |  |  |  |  |  |
| *T. theileri* K127 AJ009164 | 97.5 | 97.5 |  |  |  |  |  |  |  |  |  |  |  |  |  |  |  |  |  |  |  |
| *T. theileri* Tthc26 GQ176153 | 97.2 | 97.2 | 99.8 |  |  |  |  |  |  |  |  |  |  |  |  |  |  |  |  |  |  |
| *T.* sp. TL.AQ.40 AJ620576 | 87.3 | 87.3 | 87.2 | 87.5 |  |  |  |  |  |  |  |  |  |  |  |  |  |  |  |  |  |
| T. sp. TL.AQ.48 AJ620577 | 87.3 | 87.3 | 87.2 | 87.5 | 99.8 |  |  |  |  |  |  |  |  |  |  |  |  |  |  |  |  |
| *T.* sp. Frog ADE AJ620569 | 87.4 | 87.4 | 87.3 | 87.5 | 95.3 | 95.1 |  |  |  |  |  |  |  |  |  |  |  |  |  |  |  |
| *T.* cyclops LV492 AJ131958 | 82.2 | 82.2 | 82.2 | 82.4 | 89.6 | 89.3 | 88.1 |  |  |  |  |  |  |  |  |  |  |  |  |  |  |
| *T.* sp. TL.AQ.45 AJ620575 | 88.2 | 88.2 | 88.2 | 88.4 | 97.2 | 97.0 | 98.1 | 89.8 |  |  |  |  |  |  |  |  |  |  |  |  |  |
| **WPP585 MT883324** | 82.0 | 82.0 | 82.0 | 82.2 | 90.7 | 90.4 | 89.4 | 97.4 | 91.1 |  |  |  |  |  |  |  |  |  |  |  |  |
| **WPP601 MT883326** | 82.0 | 82.0 | 82.0 | 82.2 | 90.7 | 90.4 | 89.4 | 97.4 | 91.1 | 100 |  |  |  |  |  |  |  |  |  |  |  |
| **BRI111 MT883296** | 87.0 | 87.0 | 87.0 | 87.2 | 96.9 | 96.7 | 97.0 | 89.5 | 98.4 | 90.9 | 90.9 |  |  |  |  |  |  |  |  |  |  |
| *T.* sp. TL.AQ.22 AJ620574 | 82.0 | 82.0 | 82.0 | 82.2 | 89.8 | 89.6 | 88.8 | 97.2 | 90.5 | 98.5 | 98.5 | 90.2 |  |  |  |  |  |  |  |  |  |
| *T.* sp. TL.AV.44 cl157 AJ620573 | 87.2 | 87.2 | 87.1 | 87.4 | 95.6 | 95.4 | 98.4 | 88.8 | 98.4 | 89.7 | 89.7 | 97.2 | 89.1 |  |  |  |  |  |  |  |  |
| *T.* sp. TL.SL.1 AJ620578 | 86.8 | 86.8 | 86.7 | 86.9 | 95.4 | 95.2 | 98.1 | 88.2 | 98.1 | 89.5 | 89.5 | 97.0 | 88.9 | 99.3 |  |  |  |  |  |  |  |
| *T.* sp. ABF AJ620564 | 87.5 | 87.5 | 87.5 | 87.7 | 97.7 | 97.4 | 97.4 | 90.4 | 98.8 | 90.9 | 90.9 | 98.1 | 90.7 | 97.2 | 97.0 |  |  |  |  |  |  |
| *T.* sp. TL.AV.44 cl158b AJ620572 | 87.1 | 87.1 | 87.0 | 87.2 | 95.4 | 95.2 | 97.2 | 88.3 | 98.2 | 89.6 | 89.6 | 96.5 | 89.0 | 98.4 | 98.2 | 97.0 |  |  |  |  |  |
| *T.* sp. TL.AV.43 cl100b AJ620570 | 88.2 | 88.2 | 88.2 | 88.4 | 95.6 | 95.3 | 96.7 | 88.7 | 98.4 | 89.6 | 89.6 | 96.7 | 89.0 | 97.4 | 96.8 | 97.2 | 97.0 |  |  |  |  |
| *T.* sp. TL.AV.43 cl100e AJ620571 | 88.7 | 88.7 | 88.6 | 88.8 | 96.3 | 96.0 | 97.4 | 89.0 | 99.1 | 90.3 | 90.3 | 97.4 | 89.7 | 97.7 | 97.5 | 97.9 | 97.7 | 99.3 |  |  |  |
| *T.* sp. wallaby 10 AJ620563 | 88.4 | 88.4 | 88.4 | 88.6 | 96.0 | 95.8 | 97.2 | 88.7 | 98.8 | 90.0 | 90.0 | 97.2 | 89.4 | 97.4 | 97.2 | 97.7 | 97.5 | 99.1 | 99.8 |  |  |
| *T.* sp. TL.NG.1 AJ620581 | 90.5 | 90.5 | 90.0 | 90.0 | 96.0 | 95.7 | 96.8 | 87.5 | 98.7 | 88.7 | 88.7 | 96.8 | 88.0 | 97.1 | 96.8 | 97.6 | 97.4 | 98.9 | 99.7 | 100 |  |
