# Supplementary material for: Blood Parasites in Endangered Wildlife-Trypanosomes Discovered during a Survey of Haemoprotozoa from the Tasmanian Devil
Source: Pathogens. 2020 Oct 23;9(11):873. doi: 10.3390/pathogens9110873 (PMC7690708; doi:10.3390/pathogens9110873)
Supplement: Supplementary file 1 [file pathogens-09-00873-s001.zip › Supplementary_Tables/Supplementary_table_A1.docx]

**Supplementary Table A1.** *Trypanosoma* sequences used in phylogenetic analysis in the present study. Sequences produced in the present study indicated in bold. ^ denotes short 18S rRNA sequences only, included for comparison within the *Trypanosoma cyclops* clade (Figure 3).

| **Species** | **Isolate** | **Host** | **Location** | **18S rRNA** | **gGAPDH** |
| --- | --- | --- | --- | --- | --- |
| *T. copemani* | JD-2008a Mika | Koala (*Phascolarctos cinereus*) | NSW, Australia | - | GU966585 |
| *T. copemani* | JD-2008a Charlton | Koala (*Phascolarctos cinereus*) | QLD, Australia | GU966588 | - |
| ***T. copemani*** | **TD-BRI115** | **Tasmanian devil (Sarcophilus harrisii)** | **TAS, Australia** | **MT883297** | **MT514664** |
| *T. copemani* | H26 | Wombat (*Vombatus ursinus*) | VIC, Australia | AJ009169 |  |
| *T. copemani* | G1 | Woylie (*Bettongia penicillata*) | WA, Australia | KC753530 | KC812982 |
| *T. copemani* | APP | Wombat (*Vombatus ursinus*) | VIC, Australia | AJ620558 | AJ620277 |
| *T. copemani* | G2 | Woylie (*Bettongia penicillata*) | WA, Australia | KC753531 | KC812983 |
| *T. copemani* | Q2088 | Quokka (*Setonix brachyurus*) | WA, Australia | HQ267094 | HQ267095 |
| *T. vegrandis* | G4 | Woylie (*Bettongia penicillata*) | WA, Australia | KC753532 | KC812985 |
| *T. vegrandis* | G7 | Woylie (*Bettongia penicillata*) | WA, Australia | KC753536 | KC812987 |
| *T. vegrandis* | G5 | Woylie (*Bettongia penicillata*) | WA, Australia | KC753534 | KC812986 |
| *T. vegrandis* | G3 | Woylie (*Bettongia penicillata*) | WA, Australia | KC753533 | KC812984 |
| *T. vegrandis* | G6 | Woylie (*Bettongia penicillata*) | WA, Australia | KC753535 | - |
| *T. gilletti* | Lanie | Koala (*Phascolarctos cinereus)* | QLD, Australia | GU966589 | GU966587 |
| *T. cruzi* | G | Opossum (*Didelphis marsupialis*) | Brazil | AF239981 | GQ140351 |
| *T. cruzi marinkellei* | TryCC 344 | Bat (*Carollia perspicillata*) | Brazil | FJ001664 | GQ140360 |
| *T. erneyi* | TCC1294 | Bat (*Tadarida* sp.) | Mozambique | JN040988 | JN040965 |
| *T. dionisii* | TryCC 211 | Bat (*Eptesicus brasiliensis*) | Brazil | FJ001666 | GQ140362 |
| *T. rangeli* | AM80 | Human (*Homo sapiens*) | Brazil | AY491766 | JN040973 |
| *T. noyesi* | H25 | Kangaroo (*Macropus giganteus*) | VIC, Australia | AJ009168 | AJ620276 |
| *T. noyesi* | WC6218 | Woylie (*Bettongia penicillata*) | WA, Australia | KU354263 | KU354264 |
| *T. livingstonei* | TCC1270 | Bat (*Rhinolophus landeri*) | Mozambique | KF192979 | KF192958 |
| *T. lewisi* | Molteno B3 | Rat (*Rattus* sp.) | England | AJ009156 | AJ629272 |
| *T. microti* | TRL132 | Vole (*Microtis agrestis*) | England | AJ009158 | AJ620273 |
| ***T.* sp.** | **TD-WPP601 (genotype A)** | **Tasmanian devil (*Sarcophilus harrisii*)** | **TAS, Australia** | **MT883326** | **MT514665** |
| ***T.* sp.** | **TD-WPP602 (genotype A)** | **Tasmanian devil (*Sarcophilus harrisii*)** | **TAS, Australia** | **-** | **MT514666** |
| ***T.* sp.** | **TD-WPP585 (genotype A)** | **Tasmanian devil (*Sarcophilus harrisii*)** | **TAS, Australia** | **MT883324** | **-** |
| ***T.* sp.** | **TD-BRI111 (genotype B)** | **Tasmanian devil (*Sarcophilus harrisii*)** | **TAS, Australia** | **MT883296** | **-** |
| ***T.* sp.** | **TD-TKN211 (genotype B)** | **Tasmanian devil (*Sarcophilus harrisii*)** | **TAS, Australia** | **MT883322** | **-** |
| *T.* sp. | ABF (wallaby) | Swamp wallaby (*Wallabia bicolor*) | VIC, Australia | AJ620564 | AJ620278 |
| *T. cyclops* | LV492 | Macaque (*Macaca* sp.) | Malaysia | AJ131958 | FJ649493 |
| *T.* sp. | TL.AQ.22 | Leech (*Philaemon*) | QLD, Australia | AJ620574 | AJ620280 |
| *T.* sp. | TL.AQ.45 | Leech (*Philaemon*) | QLD, Australia | AJ620575 | - |
| *T.* sp. | TL.AV.44. cl157 | Leech (*Micobdella*) | VIC, Australia | AJ620573 | - |
| *T.* sp. | TL.AV.44 cl. 156B | Leech (*Micobdella*) | VIC, Australia | AJ620572 | - |
| *T.* sp. | TL.AV.43 cl100B | Leech (*Micobdella*) | VIC, Australia | AJ620570 | - |
| *T.* sp. | TL.AV.43 cl.101E | Leech (*Micobdella*) | VIC, Australia | AJ620571 | - |
| *T.* sp. | TL.AQ.40 | Leech (*Philaemon*) | QLD, Australia | AJ620576^ | - |
| *T.* sp. | TL.AQ.48 | Leech (*Philaemon*) | QLD, Australia | AJ620577^ | - |
| *T.* sp. | Frog ADE | Frog (*Mixophyes flaeyi*) | VIC, Australia | AJ620569^ | - |
| *T.* sp. | TL.SL.1 | Leech (*Haemadipsa zeylanica*) | Sri Lanka | AJ620578^ | - |
| *T.* sp. | Wallaby 10 | Brush-tailed rock-wallaby (*Petrogale penicillate*) | VIC, Australia | AJ620563^ | - |
| *T.* sp. | TL.NG.1 | Leech (*Leiobdella jawarerensis*) | Papua New Guinea | AJ620581^ | - |
| *T. theileri* | K127 | Cattle (*Bos taurus*) | Germany | AJ009164 | AJ620282 |
| *T. theileri* | KM | Cattle (*Bos taurus*) | Japan | AB007814^ | - |
| *T. theileri* | Bb756 | European bison (*Bison bonasus*) | Poland | KF765801^ | - |
| *T. theileri* | Tthc26 | Cattle (*Bos* sp.) | Brazil | GQ176153^ | - |
| *T. irwini* |  | Koala (*Phascolarctos cinereus*) | QLD/NSW, Australia | FJ649479 | FJ649485 |
| *T. bennetti* | KT-2 | Kestrel (*Falco sparverius*) | North America | AJ223562 | FJ649486 |
| *T.* sp. | ATT | Currawong (*Strepera* sp.) | Vic, Australia | AJ620557 | AJ620264 |
| *T. mega* | ATCC 30038 | Toad (*Bufo regularis*) | Africa | AJ009157 | AJ620253 |
| *T. rotatorium* | B2-II | Bullfrog (*Rana catesbeiana*) | Canada | AJ009161 | AJ620256 |
| *T. binneyi* | AWW | Platypus (*Ornithorhynchus anatinus*) | VIC, Australia | AJ620565 | AJ620266 |
| *T. granulosum* | UK | Eel (*Anguilla anguilla)* | Portugal | AJ620551 | - |
| *Phytomonas serpens* | N/A | N/A | N/A | U39577 | EU084892 |
| *Herpetomonas muscarum* | N/A | N/A | N/A | L18872 | DQ092548 |
